# The Effect of the Peristimulus α Phase on Visual Perception through Real-Time Phase-Locked Stimulus Presentation

**DOI:** 10.1523/ENEURO.0128-23.2023

**Published:** 2023-08-11

**Authors:** Chih-Hsin Tseng, Jyh-Horng Chen, Shen-Mou Hsu

**Affiliations:** 1 Graduate Institute of Biomedical Electronic and Bioinformatics, National Taiwan University, Taipei 10617, Taiwan (Republic of China); 2 Imaging Center for Integrated Body, Mind and Culture Research, National Taiwan University, Taipei 10617, Taiwan (Republic of China); 3 MOST AI Biomedical Research Center, Tainan City 701, Taiwan (Republic of China)

**Keywords:** α phase, MEG, perception, real time, Kalman filtering

## Abstract

The α phase has been theorized to reflect fluctuations in cortical excitability and thereby impose a cyclic influence on visual perception. Despite its appeal, this notion is not fully substantiated, as both supporting and opposing evidence has been recently reported. In contrast to previous research, this study examined the effect of the peristimulus instead of prestimulus phase on visual detection through a real-time phase-locked stimulus presentation (PLSP) approach. Specifically, we monitored phase data from magnetoencephalography (MEG) recordings over time, with a newly developed algorithm based on adaptive Kalman filtering (AKF). This information guided online presentations of masked stimuli that were phased-locked to different stages of the α cycle while healthy humans concurrently performed detection tasks. Behavioral evidence showed that the overall detection rate did not significantly vary according to the four predetermined peristimulus α phases. Nevertheless, the follow-up analyses highlighted that the phase at 90° relative to 180° likely enhanced detection. Corroborating neural parietal activity showed that early interaction between α phases and incoming stimuli orchestrated the neural representation of the hits and misses of the stimuli. This neural representation varied according to the phase and in turn shaped the behavioral outcomes. In addition to directly investigating to what extent fluctuations in perception can be ascribed to the α phases, this study suggests that phase-dependent perception is not as robust as previously presumed, and might also depend on how the stimuli are differentially processed as a result of a stimulus-phase interaction, in addition to reflecting alternations of the perceptual states between phases.

## Significance Statement

α Activity, a widely observed neural phenomenon, is postulated to be essential for organizing visual perception. However, our previous understanding of the functional relevance of the α phase is primarily inferred from the prestimulus or externally entrained phase. This study monitors real-time phase activity and presents stimuli that are phased-locked to different stages of the α cycle. This new approach allows us to investigate whether and how the α phases, during which stimuli are concurrently presented, directly lead to behavioral and neural changes in perception. Our evidence suggests that the extent to which the α phases affect perception depends on an early interaction between the phase and incoming stimuli, which is involved in shaping the perceptual fates of the stimuli.

## Introduction

The phases of rhythmic brain activity are essential for the organization of brain processes for cognitive operation (Bollimunta et al., 2011), as the flow of information could be conceived as being framed by the phase within a rhythmic cycle (Schroeder and Lakatos, 2009; Thut et al., 2012). In particular, the α phase (8–12 Hz) is thought to reflect cortical excitability (Lindsley, 1952; Fries, 2005; Klimesch et al., 2007; Haegens et al., 2011) and exerts a moment-by-moment influence on visual perception. Previous studies (Varela et al., 1981; VanRullen, 2016) have proposed that incoming perceptual information that coincides with the α phase indexing high excitability is amplified, whereas it is suppressed when coinciding with the low excitability phase. However, the idea of α phase-dependent visual perception is still not fully substantiated (Keitel et al., 2022). Although supporting evidence has shown that hits (detected stimuli) or misses (undetected stimuli) are preferentially locked to distinct α phase angles during visual detection tasks (Busch et al., 2009; Mathewson et al., 2009; Dugué et al., 2011; Helfrich et al., 2014; Jaegle and Ro, 2014), contrasting evidence has also recently accumulated, and a negative association between α phases and detection rates has been reported (Benwell et al., 2017; Ruzzoli et al., 2019; de Graaf et al., 2020; Zazio et al., 2022).

**Figure 1.**
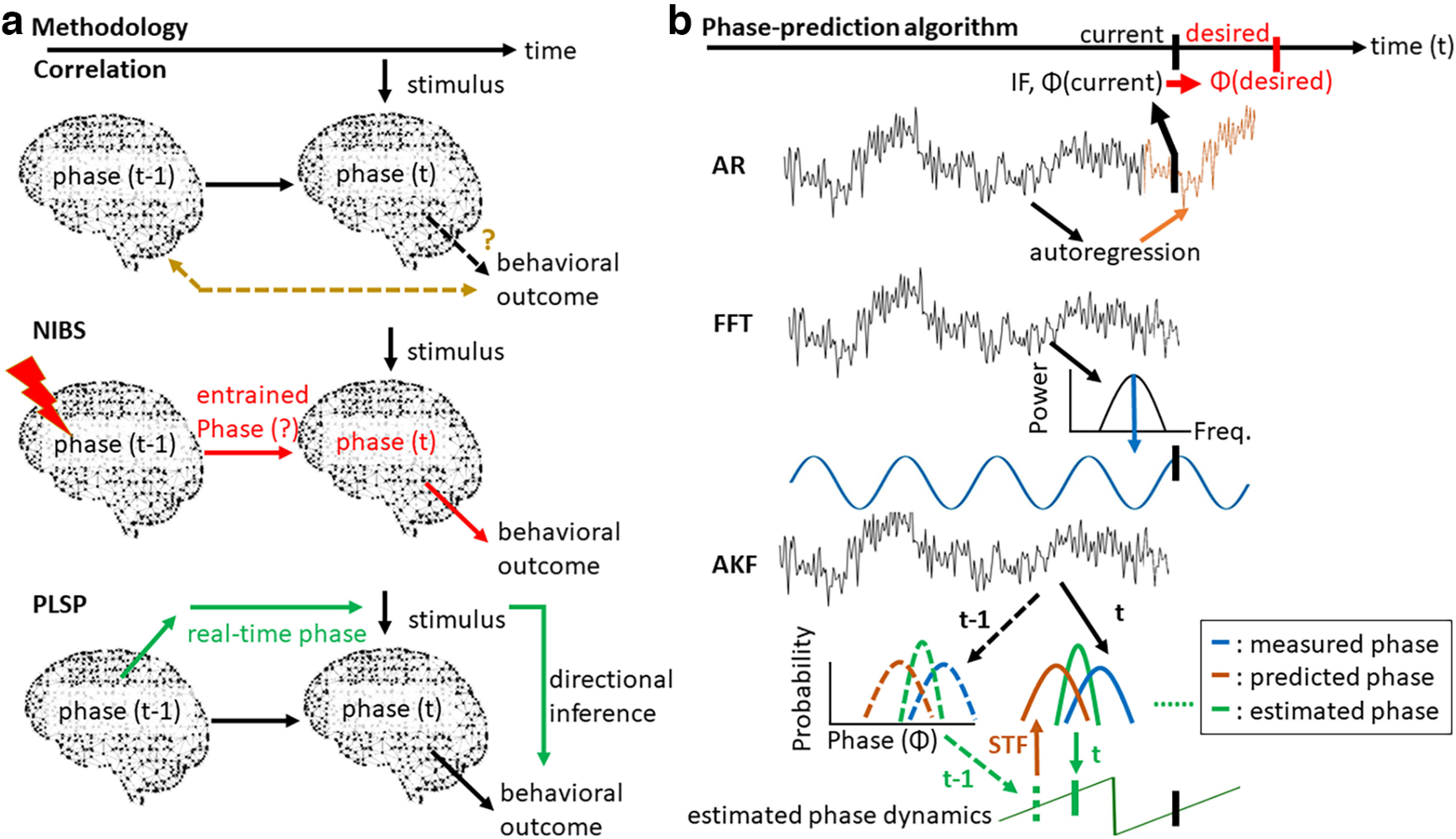
Schematic comparison between the current and previous methodologies. ***a***, To relate the peristimulus α phase with visual detection, the correlation approach (top panel), during which a stimulus sequence is arbitrarily presented, quantifies the link between behavioral outcomes and offline sorted prestimulus phases [phase(t−1)] instead of peristimulus phases [phase(t)]. This limitation reflects the constraints of window-based/acausal signal processing that typically utilizes the data extended in time. Specifically, the poststimulus phase activity elicited by the stimulus may crossover into the peristimulus window, thus obscuring direct examination of the effect of the α phase around stimulus onset. Alternatively, noninvasive brain stimulation (NIBS; middle panel) aligns the brain phase with the phase of external stimulation so that stimulus presentation can be locked to the desired phase [phase(t)] of the stimulation waveform; however, concerns regarding the efficacy of such phase alignment have been raised. The current approach instead aims to monitor the instantaneous, endogenous phase in real time to guide phase-locked stimulus presentation (PLSP; bottom panel) to provide directional inference. ***b***, To implement PLSP, the core of the algorithms is to resolve the limitations and artifacts resulting from the system and standard acausal signal processing so that phase (Φ) and instantaneous frequency (IF) at the current time point (black vertical bar) can be accurately estimated. Because the current phase actually indicates the near past phase because of a system-specific time lag, when the current phase approaches the proximity of the future desired phase (red vertical bar), a time delay between t_current_ and t_desired_ is calculated based on the estimated Φ_current_ and IF_current_. A stimulus is then presented immediately after this time delay to phase-lock to the desired phase. To estimate Φ_current_ and IF_current_, the autoregressive (AR; top panel) algorithm extracts the temporal pattern of the past signal (black curve) to construct a forward-estimated segment (orange curve). The fast Fourier transform (FFT; middle panel) projects the past signal into the frequency domain to capture the dominant frequency and then uses this information (blue curve) to perform forward phase estimation. For the current adaptive Kalman filtering (AKF; bottom panel) algorithm, a new estimated phase (green curve) is recursively formed over time, part way between the measured phase derived from the signal (blue curve) and the predicted phase (brown curve) derived from the state transition function (STF). The STF describes how phase states transition between time points (e.g., t−1 to t) and assumes that the instantaneous phase evolves with a constant IF. To improve phase estimation, the AKF algorithm adaptively favors the contribution of the predicted phase whenever the measured IF exceeds the α range because of signal noise.

The aforementioned controversy might reflect the limitations of previous approaches (Fig. 1*a*). On the one hand, the widely adopted correlation approach counterintuitively infers the detection performance based on the α phase observed before stimulus onset, which usually peaks up to −200 ms or more, rather than the critical phase where the stimulus is actually presented (Busch et al., 2009; Mathewson et al., 2009; Benwell et al., 2017; Ruzzoli et al., 2019; Zazio et al., 2022). This limitation might reflect that offline examination of the phase effect around stimulus onset is challenging because of the “smearing” effect caused by phase adjustment after stimulus presentation (Brüers and VanRullen, 2017), especially when window-based/acausal signal processing that typically utilizes the data extended in time is employed. In other words, the original peristimulus phase is unlikely to be backwardly recovered offline from the mixed phase information that is additionally composed of stimulus-driven phase dynamics and their interactions. This issue may hinder the correlation approach because this approach is primarily built on trial sorting according to the offline phase, but the peristimulus phase, when being estimated offline on a trial-by-trial basis, cannot be properly validated because of the “smearing” effect and is thereby rendered unknown. On the other hand, the noninvasive brain stimulation (NIBS) approach may hint at a causal link between the peristimulus α phase and visual detection by applying external stimulation, such as rhythmic stimulus stimulation and transcranial magnetic or alternating current stimulation; such stimulation can entrain the brain phase so that stimulus presentation is aligned with the phase of the external stimulation (Dugué et al., 2011; Helfrich et al., 2014; Jaegle and Ro, 2014; Spaak et al., 2014; de Graaf et al., 2020). However, stimulation-related artifacts may concurrently contaminate ongoing brain activity, and the magnitude of the entrainment that can be achieved between the applied stimulation and the underlying brain activity has been questioned (Bergmann and Hartwigsen, 2021; Kasten and Herrmann, 2022). Moreover, it remains unclear whether endogenous and externally driven oscillations in the visual system are functionally the same.

To address the above issues, this study employed a phase-locked stimulus presentation (PLSP) approach to examine the direct role of the peristimulus α phase that naturally occurs without an external driving force during visual detection (Zrenner et al., 2016). In principle, this approach guides stimuli presented at desired phases in real time via magnetoencephalography (MEG) recordings (Fig. 1*a*). Because real-time phase estimation was performed before actual stimulus presentation, the “smear” of poststimulus phase effects could be avoided. In addition, the relation of stimulus presentation relative to the ongoing phase was controlled and, in turn, produced specific changes in perceptual phenomena. Therefore, the NIBS and our approaches function in a similar manner, except that the phase is passively monitored in real time in the latter approach rather than actively manipulated by applying external stimulation. A few previous studies have employed the PLSP approach to investigate phase effects on reaction times based on the fast Fourier transform (FFT) algorithm (Callaway and Yeager, 1960; Dustman and Beck, 1965; Vigué-Guix et al., 2022). Nevertheless, in those studies, stimuli can be better locked to a given phase because participants responded to visual flashes with their eyes closed, and thereby, the system-specific time lag is of less concern. To implement the PLSP approach in the current but more complex experimental setting, we introduced a new phase-prediction algorithm that capitalizes on adaptive Kalman filtering (AKF; Fig. 1*b*; Simon, 2006). This algorithm adaptively and iteratively combines two sources of information, the measured phase from MEG recordings and the predicted phase from the state transition function that was created for describing instantaneous phase dynamics, to produce optimal phase estimates by minimizing variance. We showed that the AKF algorithm generally achieved a more robust phase-locked presentation than the commonly existing autoregressive (AR; Chen et al., 2013; Blackwood et al., 2018; Zrenner et al., 2020) and FFT algorithms (Mansouri et al., 2017; Rivero and Ditterich, 2021). With the newly developed algorithm, participants’ instantaneous α phase activity at the predetermined posterior parietal sensor was tracked online in a subsequent experiment, as previous research has indicated that the α phase from the posterior parietal region is crucially involved in visual detection (Mathewson et al., 2009; Helfrich et al., 2014; Jaegle and Ro, 2014). This information was used to forward inform the presentation of masked targets at four predetermined desired phases, which spanned one full α cycle from 0°, 90°, 180°, to 270°, a common choice in the NIBS approach because of practical limits on the number of trials that can be tested in an experimental session (Helfrich et al., 2014; Jaegle and Ro, 2014). Participants were instructed to concurrently detect those implicitly phase-locked targets that were adjusted at the luminance threshold before the experiment and thereby physically identical across trials. In summary, the objective of this study is twofold. First, we aimed to develop a new algorithm for better implementing real-time phase-locked stimulation. Second, we examined the extent to which the peristimulus instead of prestimulus phase enhances detection, as previously presumed, through this real-time approach.

## Materials and Methods

### The development of the AKF algorithm for PLSP

#### Data preparation

Two data types were used to evaluate algorithm performance. Synthetic data, which lasted for 5 min at a resolution of 1000 Hz, were generated using sinusoids at 8–12 Hz. White noise was added to the synthetic data, and noise amplitude was scaled to achieve a signal-to-noise ratio (SNR) level of one, as SNRs smaller than one are common in real electrophysiological data. Here, the SNR was defined in decibels of a signal by computing the ratio of its summed squared magnitude to that of the noise.

Resting-state real data were prepared from 15 right-handed participants (12 males and three females, mean age ± SD = 26.27 ± 3.22 years, range = 22–32) who rested and fixated at the center of the screen while their MEG data were recorded for 10 min. MEG recordings were performed using a 306-channel whole-head MEG system (Elekta Neuromag TRIUX) with a sampling rate of 1000 Hz. All procedures were conducted in accordance with the Declaration of Helsinki. FieldTrip (RRID:SCR_004849) and MATLAB (RRID:SCR_001622) software were used for data preprocessing, analysis, and visualization.

#### The general pipeline of PLSP

For consistency purposes, a general pipeline was used for all the algorithms compared. Approximately three cycles of the data segment, according to the frequency band of interest (i.e., 300 ms for the α band), were extracted from the start of the synthetic or MEG signal. The data point at the end of the data segment was regarded as the “current” time point t_current_ (Fig. 1*b*). This window size was chosen to ensure that the oscillatory characteristics of the data could be captured while achieving a good balance between computation speed and performance (Mansouri et al., 2017). Next, the extracted data were zero-phase bandpass filtered [finite impulse response (FIR) filter, order = three times the lower frequency bound, bandwidth = 8–12 Hz]. Filtered data were then submitted to each algorithm (see below) to estimate the instantaneous phase (Φ) and frequency (IF) at t_current_. To correct for the system lag that occurred during real-time implementation because of data, hardware (including stimulation) and real-time sampling processing, the phase/time delay between t_current_ and t_desired_ (Fig. 1*b*) was considered. The relationship between the phase/time delay, the current instantaneous phase and frequency can be described as t_desired-current_ = sampling rate * (Φ_desired_ – Φ_current_)/(2 * π * IF_current_). When the instantaneous phase approached the proximity of the future desired phase (here, we investigated 45° or 180° of the phase delay), a marker (for synthetic data)/trigger pulse (for resting-state MEG data) was labeled/sent immediately to phase-lock a given desired phase. Here, the desired phase was predefined at 0°, 90°, 180°, or 270° to cover a full α cycle. To mimic the later real-time MEG experiment, incoming data were consecutively read in chunks of 30 samples, and thereby, the instantaneous phase and frequency were updated every 30 samples. The whole procedure described above was repeated until the end of the signal.

#### AR algorithm

After removing the edges (75 ms from both ends according to (Chen et al., 2013) and our own pilot simulation) of the filtered data, the remaining segment was submitted to the AR model of order **p**:

$$x_{t}=\alpha_{0}+\overset{p}{\underset{k=1}{\sum }}\alpha_{k}x_{t-k}+\varepsilon_{t},$$

where **α** is the model coefficient, **α**_0_ is a constant and **ε** is white noise. The model order was adaptively adjusted according to the Bayesian information criterion, and the Yule–Walker method was adopted to estimate the coefficients (Chen et al., 2013). Using the obtained AR coefficient, the future signal segment was iteratively forward-predicted to encompass t_current_ until 75 ms after t_current_. Next, the Hilbert transform was applied to derive the instantaneous phase and frequency.

#### FFT algorithm

The filtered data were first zero-padded to 1800 sample points to increase the frequency resolution. Next, the FFT of the signal segment was calculated to determine the dominant frequency that had the maximum power in the signal. The instantaneous phase and frequency of the dominant component at t_current_ were extracted and used to forecast the time delay from the forthcoming desired phase.

#### AKF algorithm

A new estimated phase was recursively formed over time, starting from the 1/2 frequency cycle before t_current_, part way between the measured phase derived from the Hilbert-transformed signal and the predicted phase derived from the state transition function. The function describes how phase states transition between time points where the phase evolves with a constant instantaneous frequency. In the algorithm, Gaussian distributions were used to represent the state variables and their error. This assumption is regarded as valid here, as the phase is typically represented in terms of the von Mises distribution, which is the circular analog of the normal distribution. As further described below, to reduce the edge effect and phase singularity, an adaptive parameter was additionally introduced, such that at each time step, the AKF algorithm adaptively favored the contribution of the predicted phase whenever the measured instantaneous frequency exceeded the α range because of signal noise. In summary, the AKF algorithm adaptively combined two sources of information, predicted states and noisy measurements, to produce optimal estimated states by minimizing their variance. The algorithm is formally formulated as described below.

Prediction step:

1. A transition function was used to predict the state at the next time step by
$${\hat{x}}_{t|t-1}=F_{t}x_{t-1|t-1},$$
  where ${\hat{x}}_{t|t-1}$ denotes the estimate of state **x (** $x={\left[x\dot{x}\right]}^{T}$, *x*: instantaneous phase; $\dot{x}$: instantaneous frequency) at time t given state **x** at time t−1. Because the algorithm was implemented in a short time window, we assumed that no acceleration occurred, and thereby, the state transition function was formulated as $F=\left[\begin{array}{cc} 1 & \Delta t\\ 0 & 1 \end{array}\right]$ (Δt: time difference) to describe the phase dynamics.
2. The state covariance **P** was adjusted to account for the uncertainty in prediction by
$${\hat{P}}_{t|t-1}=\alpha^{2}F_{t}P_{t-1|t-1}F_{t}^{T}+Q_{t},$$

where **Q** represents the process noise covariance. Because the algorithm was performed within a very short time window, acceleration was assumed to be locally constant, and in turn, the initial **Q** was set to 0. We initialized **P** with

$$P=\left[\begin{array}{cc} R_{0} & 0\\ 0 & {\dot{x}}_{\text{max}}^{2} \end{array}\right].$$

The diagonal of **P** contains the variance in each state variable. **R**_0_ is an initial variance in phase and was set to 93̂2 (degree) based on the SD of the phase differences between the synthetic data and its ground truth. The maximum velocity squared is the initial variance for the instantaneous frequency and was set to 4.32̂2 (degree/ms), which was converted from 12 Hz, the upper limit of the α band. Here, we introduced a parameter **α** to mitigate the edge effect and phase singularity (Hurtado et al., 2004; Mortezapouraghdam et al., 2018). In practice, **α** was set to 1 but adaptively became 0.8 whenever the measured instantaneous frequency exceeded the range of the α band.

Update step:

1. The residual **y** between the predicted state and the measurement is computed by
$$y_{t}=z_{t}-H_{t}{\hat{x}}_{t|t-1},$$
  where **z** is the phase measurement from the filtered signal at the predetermined sensor, and **H** $\left(H=\left[\begin{array}{cc} 1 & 0 \end{array}\right]\right)$ is the measurement function.
2. The Kalman gain **K**, which scales the uncertainty (i.e., covariance) from the measurement and state prediction, is computed by
$$K_{t}={\hat{P}}_{t|t-1}H_{t}^{T}{\left(H_{t}{\hat{P}}_{t|t-1}H_{t}^{T}+R_{t}\right)}^{-1},$$
  where **R** is a measurement of noise covariance and was set to 93̂2, the same as **R**_0_. **K** is bounded between 0 and 1. A larger **K** represents a greater contribution from the measurement and vice versa.
3. The state was updated based on the Kalman gain by
$${\hat{x}}_{t|t}={\hat{x}}_{t|t-1}+K_{t}y_{t}$$
4. The state covariance was updated by
$$P_{t|t}=(I-K_{t}H_{t}){\hat{P}}_{t|t-1},$$
  where **I** is an identity matrix. In practice, to ensure numerical stability, the Joseph equation was used instead
$$P_{t|t}=\left(I-K_{t}H_{t}\right){\hat{P}}_{t|t-1}{\left(I-K_{t}H_{t}\right)}^{T}+K_{t}R_{t}K_{t}.$$

### Assessment of algorithm performance for PLSP

The performance of the algorithm was assessed offline by comparing the predicted desired phase, which was marked by the marker/trigger as being determined by the algorithm, with the predetermined desired phase (0°, 90°, 180°, or 270°). To obtain the predicted desired phase, we bandpass filtered the MEG data with a zero-phase FIR filter (order = three times the lower frequency bound, bandwidth = 8–12 Hz). This step was valid given that the “ground-truth” phases in the artificial and resting-state, stimulus-free MEG data could be obtained or reasonably estimated. Then, the filtered data were Hilbert transformed to extract the phase value at the trigger point. Performance accuracy was quantified using the circular mean to measure the average of the absolute phase distances between the estimated and desired phases over “stimulus presentations” (i.e., the numbers of triggers). We also quantified performance precision, i.e., the spread of the phase distances, by calculating their circular SD. Small accuracy or precision values indicate that the phases estimated by the algorithm are close to the desired phases or more concentrated (high certainty).

### Phase-dependent visual perception experiment

#### Participants

According to previous results using a similar task design (Mathewson et al., 2009), the effect size of Cohen’s *d* was estimated to be 1.3 based on the reported paired *t* test, which is the analysis used to examine the difference in the detection rates between the two phase groups on the high α power trials in the original paper. Notably, this effect size is biased and may be underpowered, as the current analysis path is different from that conducted in the prior study. Because an accurate estimation of the sample size required for the current new approach is difficult, we aimed for a higher power estimate of 95%, relative to the convention of 80%, with an α = 0.01. To reach this level of power with the above effect size as an indirect approximation, a new group of 15 participants was recruited (nine males, mean age ± SD = 29.73 ± 11.91 years, range = 20–62 years) using G*Power (RRID:SCR_013726). (Correction: our reported effect size was miscalculated because the t statistical value was misidentified as 4.3. In fact, the effect size should be 1.37 based on *t*_(10)_ = 4.53 in the original paper. As a result, the required sample size should be 14.) All the enrolled participants had normal or corrected-to-normal vision, no past neurologic or psychiatric history and provided written informed consent. All procedures were conducted in accordance with the Declaration of Helsinki.

#### Task

Each trial began with the presentation of a black fixation cross at the center of the screen (refresh rate = 60 Hz) for 250 ms, followed by a jittered blank screen (mean ± SD = 882 ± 111 ms across participants). This jitter was naturally introduced by the analysis pipeline, reflecting the decision timing pertaining to whether the instantaneous phase approached the proximity of the desired phase. The durations of the jitter (0°: 887 ± 132 ms; 90°: 879 ± 121 ms; 180°: 873 ± 129 ms; 270°: 892 ± 107 ms) were not significantly different across the four desired phases (one-way repeated-measures ANOVA, *F*_(3,42)_ = 0.29, *p* = 0.832). A dark gray target disk was then presented for 16.6 ms with a 50 ms interstimulus interval (ISI) before the dark gray annulus mask appeared for 33.3 ms. All stimuli used in the experiment were black (0 cd/m^2^), displayed on a gray background. Before the experiment, the luminance threshold of the background was established for each participant. This calibration session was designed to ensure no ceiling or floor performance in the subsequent real-time experiment. The calibration session included randomly interleaved psychophysical staircases. A one-up, one-down staircase procedure was employed to converge to a detection rate of ∼50%. This procedure was terminated after five reversals. The target disk and the annual mask subtended a visual angle of 1° and 2° from the screen center. Subjects had 1520 ms to indicate whether they had seen the target. On average, participants failed to respond on 4% of trials, and data from these trials were excluded from further analysis.

Each participant completed 16 runs. Each run consisted of 72 trials, with half of the trials containing both the target and mask, a quarter of the trials containing the mask and a blank screen in place of the target, and the remaining quarter of the trials containing the target without the mask. To acquaint participants with the procedure, the experiment began with practice trials in the same proportion of the trial types. To ensure a sufficient number of trials for each behavioral outcome, the mask intensities were adjusted for individual participants before the experiment to obtain the luminance that yielded the target less detectable.

#### MEG data recordings

MEG recordings were performed identically using a 306-channel whole-head MEG system (Elekta Neuromag TRIUX) with a sampling rate of 1000 Hz. Eye-related activities were monitored via vertical and horizontal electrooculography. Electrocardiography (ECG) electrodes were placed over the chest close to the left and right clavicles.

#### Real-time stimulus presentation

Real-time MEG data were acquired using rtMEG (Sudre et al., 2011) in chunks of 30 samples because of system limitations. No additional noise reduction, such as signal source separation, was applied to the data. The phase-locked stimulation procedure was identical to that previously described and implemented online. In short, the data were zero-phase bandpass filtered at 8–12 Hz (FIR filter, order = three times the lower frequency bound), and the instantaneous α phase and frequency were estimated using the AKF algorithm. When the current instantaneous phase was 180° from the future desired phase, the upcoming stimulus (target-only and masked-target trials)/trigger (mask-only trials) delivery was scheduled. Each desired phase condition had four runs. Within each run, trials were blocked by a given desired phase to facilitate algorithm computation.

#### Offline MEG data preprocessing and event-related field (ERF) analysis

Continuous MEG data from the planar gradiometer sensors were segmented into 2000-ms epochs starting from 1000 ms before target onset. Trials contaminated with muscular artifacts (over 100 Hz) were visually identified and rejected. Eye movements, eye blinks, and cardiac artifacts were removed using independent component analysis (three to four components removed). After preprocessing, there were 563 ± 18 (mean ± SD; 0° desired phase: 141 ± 4, 90°: 141 ± 4, 180°: 141 ± 6, 270°: 140 ± 5) masked-target trials, 281 ± 10 (0°: 70 ± 3, 90°: 71 ± 2, 180°: 70 ± 3, 270°: 70 ± 2) target-only trials, and 281 ± 9 (0°: 70 ± 3, 90°: 71 ± 2, 180°: 70 ± 3, 270°: 70 ± 2) mask-only trials. For the ERF analysis, the data were averaged across trials for each phase condition and participant. The averaged data were baseline-corrected by subtracting the mean activity during the baseline period (200–0 ms preceding target onset).

#### Offline time frequency, power, and instantaneous frequency analysis

The amplitude and phase at each time-frequency point were extracted using the Hilbert transform. Before the transform, the data were zero-phase bandpass filtered (FIR filter, order = three times the lower frequency bound) to create 5 frequency steps, with center frequencies from 8 to 12 Hz and a bandwidth of 2 Hz. The power activities were obtained by averaging the spectral amplitude across trials for each experimental condition and participant. The averaged power activities for each data point were normalized to a baseline ranging from 300 to 600 ms preceding target onset. Normalization in decibels involved calculating the 10log10 transform of the power relative to the mean baseline power on a frequency-by-frequency basis.

To obtain the prestimulus instantaneous frequency (Samaha and Postle, 2015), the data were zero-phase bandpass filtered at 8–12 Hz (FIR filter, order = three times the lower frequency bound). Phase angle time series were extracted from the filtered data with the Hilbert transform. The temporal derivative of the phase angle time series describes how the phase changes over time and thus corresponds to the instantaneous frequency in Hertz (when scaled by the sampling rate and 2π). The instantaneous frequency was filtered with a moving median filter with a window of 10 ms before averaging across trials to mitigate noise in the phase angle time series without distorting the data.

#### Offline phase coherence analysis

To quantify the degree of phase clustering (or phase locking) in response to the stimuli, we computed the cosine-similarity version of intertrial phase coherence (ITC_CS_; Chou and Hsu, 2018), as this metric is robust to sample-size bias compared with the conventional one. ITC_CS_ represents the mean cosine of the angles of all phase pairs from any two trials $\theta_{i},\theta_{j}$. For a given sensor *s*, frequency *f*, time *t*, and a total number of trials *N*,

$$ITC_{cs}\left(s,f,t\right)=\frac{2}{N\left(N-1\right)}\overset{N-1}{\underset{i=1}{\sum }}\overset{N}{\underset{j=i+1}{\sum }}\cos\left(\theta_{i}-\theta_{j}\right).$$

An ITC_CS_ close to 1 reflects strong unimodal phase clustering (i.e., all trials exhibit the same phase), whereas an ITC_CS_ close to 0 reflects low phase clustering (i.e., the distribution of phases across trials is uniform). An ITC_CS_ can also be negative whenever a group of two phases are distant from each other over 90°, which would lead to negative cosine similarity.

#### Surrogate data

To assess whether stimulus presentation elicited phase coherence at the group level, we estimated chance-level phase coherence for each participant at every time-frequency point, which was later compared with the original coherence using cluster-based permutation testing. Because phase coherence represents the timing of phase activity locked to target onset over trials, surrogate data were created using a cut-and-swap strategy to disrupt the relationship between stimulus onset and phase activity while minimizing the distortion of phase dynamics (Hurtado et al., 2004). Specifically, we randomly selected a single time point and exchanged the resulting two sections of data in each MEG trial to recompute surrogate ITC_CS_. This procedure was repeated 1000 times, resulting in a distribution of surrogate ITC_CS_ values for each participant, time, and frequency. For each time-frequency point, we defined the chance level as the mean of the surrogate values (e.g., see (Dugué et al., 2011) for a similar approach).

#### Jackknife estimate of onset and offset latency

For each participant, onset or offset latency was defined as the time when the front or the back end of the waveform of interest reached 30% of its peak amplitude (Liesefeld, 2018). With this approach, a jackknife latency estimate was scored for each of 15 grand average waveforms. Each of these grand average waveforms was computed from a subsample of 14 of the 15 participants (i.e., one participant was omitted from one of the subsample grand averages). Last, the mean of those 15 jackknife estimates was reported as the grand estimate.

#### Cluster-based permutation test

Cluster-based permutation tests, implemented in Fieldtrip, were conducted to determine if the data differed significantly between conditions at the group level. This statistical test does not require specific assumptions about the shape of the population distribution and controls for the problem of multiple comparisons (Maris and Oostenveld, 2007). In these tests, conditional differences were quantified by means of paired *t* tests for every time period or time-frequency sample. The samples with t values exceeding the threshold (*p* < 0.05, two-tailed) were clustered into connected sets based on temporal or temporal-frequency adjacency. The cluster with the maximum sum of t values was used as a test statistic. A distribution was then generated by randomly permuting the data across the conditions for each participant and recalculating the test statistic using a Monte Carlo estimate after repeating the calculation 1000 times. Finally, two-tailed *p* values were determined by evaluating the proportion of the distribution resulting in a test t statistic larger than the observed t statistic.

#### Representational similarity analysis

To relate the ERFs obtained from the masked-target trials with those from the mask-/target-only trials, we conducted representational similarity analysis (RSA) using the RSA toolbox (Nili et al., 2014). For individual participants and trial types (masked-target trial vs masked-/target-only trial), ERFs ranging from 70 to 121 ms after stimulus onset were extracted for each desired phase (90° and 180°) and behavioral outcome (hit and miss). For every phase-behavioral outcome pair, we computed cosine similarity and stored the result in a 4 × 4 symmetric matrix. We then converted these similarity indices into a dissimilarity measure (1, the cosine of the included angles), which was rank-transformed and scaled so that it was bound between 0 (no dissimilarity) and 1 (complete dissimilarity). The representational dissimilarity matrices (RDMs) obtained from the masked-target trials were defined as a reference and averaged across subjects (a similar result was found when the RDMs from masked-/target-only trials were used as a reference). Next, Kendall’s rank correlation was calculated between the average masked-target RDMs and the subject-specific masked-/target-only RDMs. The relatedness between these two types of RDMs was tested using a one-sided signed-rank test across the single-subject RDM correlations.

#### Sinusoidal fitting

The oscillatory features of the data were characterized by fitting the data with a sine function:

y(t)=asin(2πft + Φ) + b + c,

where y is the data, a is the amplitude, f is the frequency, t is time, Φ is the phase shift, b is the offset and c is the noise term. For fitting the data, the offset was first estimated by the mean of all y values. FFT was applied to y to provide initial values of f, a, and Φ for regression. The ultimate f and Φ were obtained whereby the summed squares of the residuals were minimized.

#### Behavioral decision model fitting

To test whether information content in the early ERFs played a role in shaping ultimate detection rates, we trained a logistic regression model to classify behavioral hits (seen targets) and misses (unseen targets) from z-transformed single-trial MEG data during the significant time period (70–121 ms). To ensure reliability and generalizability of classification, repeated stratified fivefold cross-validation was conducted. Specifically, within each fold, training was performed on a randomly selected subset of 80% of trials. The trained classifier was then tested to predict the behavioral outcomes of the remaining 20% of trials. Notably, stratification was used to ensure that each outcome was approximately equally represented in each fold. The above stratified fivefold cross-validation was repeated 10 times with different randomization in each repetition to improve the estimated performance. The resulting averaged performances were reported. All these analyses were conducted with Scikit–Learn packages (RRID:SCR_002577).

### Statistics

For circular data, the Watson–Williams test was used to examine the significant difference between the means, the Harrison–Kanji test was used to examine the main and interaction effects on two-factor data, and circular-circular correlation was used to assess the correlation between two variables. All these tests were performed using the CircStat toolbox (RRID:SCR_016651). For behavioral data, one-way repeated-measures ANOVA and paired *t* tests were adopted. For neural data, the cluster-based permutation test was used to control for the problem of multiple comparisons. Two-way repeated-measures ANOVA and *t* tests were used for the follow-up analysis. A signed-rank test was conducted for RSA. For extremely small *p* values (*p* < 0.0001), *p* = 0.0001 was reported.

### Data and code availability

All preprocessed data necessary to reproduce the figures are available at https://osf.io/kfpbd/?view_only=1a688b60444443fcab9ff1c63a9daa06. All the codes necessary to reproduce the figures and to perform the AKF algorithm are available at https://osf.io/kfpbd/?view_only=1a688b60444443fcab9ff1c63a9daa06. The original data can be shared by the corresponding author on request if data privacy can be guaranteed.

## Results

### The performance of the AKF algorithm

**Figure 2.**
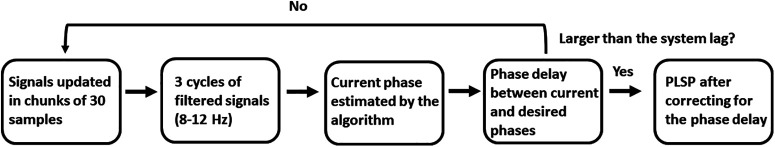
The general pipeline of phase-locked stimulus presentation (PLSP). Incoming data were consecutively read in chunks of 30 samples (30 ms on average) because of system limitations using rtMEG (Sudre et al., 2011). Three α-cycles of the data segment were extracted back from the current time point and zero-phase bandpass filtered (8–12 Hz). Filtered data were then submitted to each algorithm to estimate the instantaneous phase and frequency at the current time point. To correct for the system lag that occurred during real-time implementation because of data, hardware and real-time sampling processing, the phase/time delay between the current and desired phases was continuously updated. When the phase delay equaled to 90° or 180° during simulation and to 180° during the real-time experiment, a marker (synthetic data), trigger pulse (resting-state MEG data), or a stimulus (visual detection experiment) was labeled or sent immediately to compensate for the lag so that a given desired phase was locked. When the current phase fell within the range of the prespecified phase delay, the pipeline was reinitiated because the system lag was not properly compensated.

We first evaluated the performance of the proposed AKF algorithm against that of the AR and FFT algorithms in a simulated real-time setting that was later implemented in a visual detection experiment. We used both synthetic data and resting-state MEG data. The latter was acquired at the predetermined posterior parietal sensor with identical real-time parameters except that no stimulus was sent. For consistency purposes, a general pipeline of PLSP was used for all the algorithms compared (Fig. 2; for details, see Materials and Methods, The general pipeline of PLSP). In principle, the current instantaneous phase was estimated by the algorithm after data sampling, filtering (α band 8–12 Hz) and extraction. Notably, the current phase actually reflected the near past phase because of the system-specific time lag during real-time implementation because of data, hardware (including stimulation) and sampling processing. To correct for this lag, PLSP must be implemented in advance whenever the phase/time delay between the current and future desired phases approximately equaled to the system lag. During the simulation, we therefore included this factor for examination, which is expressed in terms of the phase/time delay between the current instantaneous phase and the future desired phase. We investigated the phase delay at two points: 45° and 180°. The latter value was used in the subsequent real-time experiment because the system lag was ∼50 ms with the current settings. The desired phases predicted by the algorithms were compared offline with the actual desired phases in terms of accuracy (circular mean of the absolute phase distances between the two phases) and precision (circular SD of the absolute phase distances). Here, the actual desired phases were predetermined a priori (0°, 90°, 180°, or 270°), whereas the predicted desired phases were marked by the algorithm-determined markers/triggers, and their values at the markers/trigger points were extracted from the offline filter-Hilbert transform on the data epoch. Smaller values of accuracy or precision indicated that the phases estimated by the algorithm were close to the desired phases or more concentrated (high certainty).

**Figure 3.**
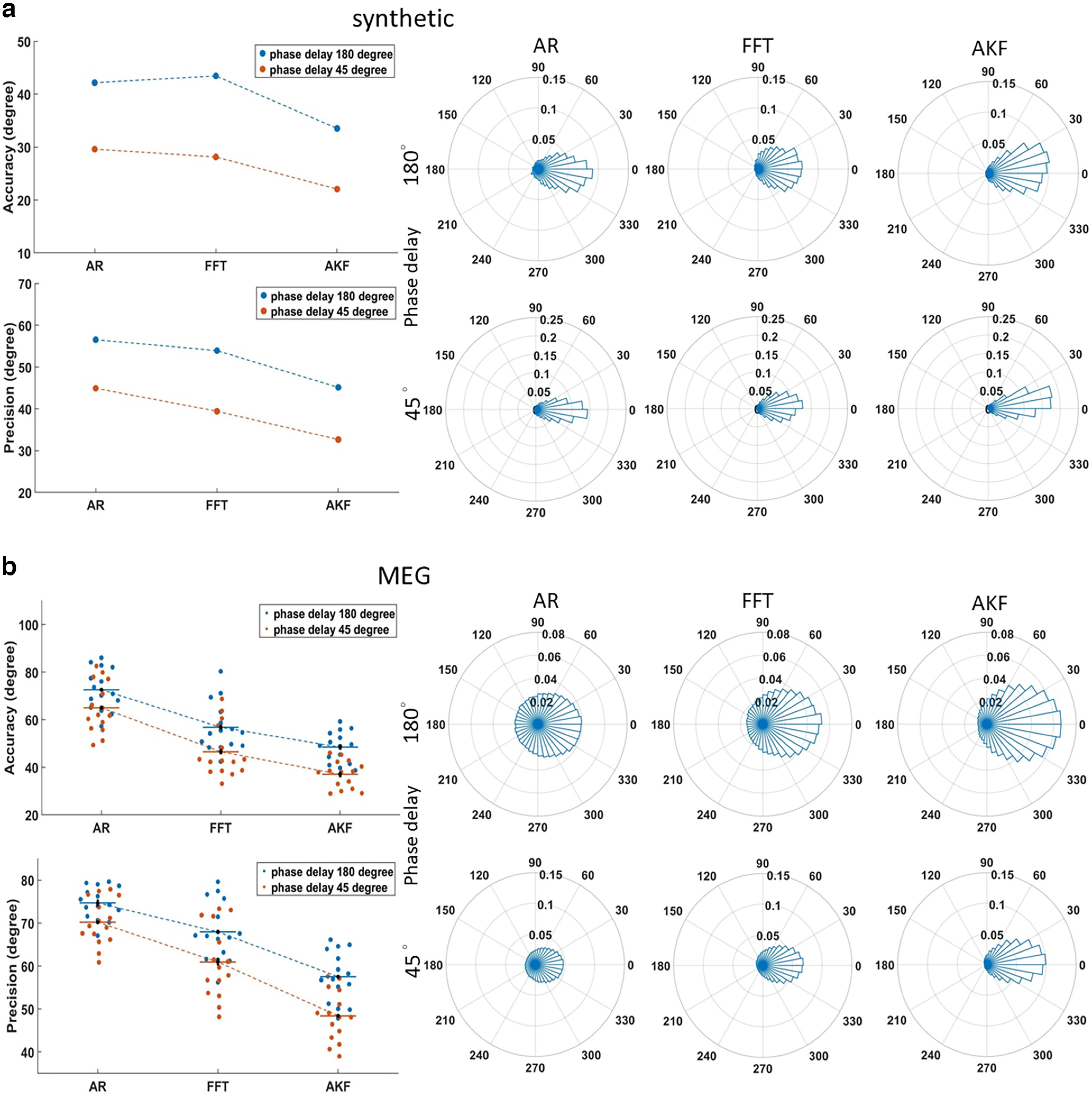
Algorithm performance during simulated phase-locked stimulus presentation (PLSP). ***a***, Synthetic data. The right panels show the circular histograms of the phase distances between the estimated and predetermined desired α phases collapsed across all four phase conditions for the 90° and 180° phase delays. The distance from the origin indicates the number (1000×) of presentations (estimated desired phases) falling within a bin. The left panels display the accuracy (i.e., the absolute phase distances from the desired phases) and precision (i.e., the spread of the absolute phase distances) as a function of the phase delay and the algorithm. Small accuracy or precision values indicate that the phases estimated by the algorithm are close to the desired phases or more concentrated (high certainty). AR: autoregressive; FFT: fast Fourier transform; AKF: adaptive Kalman filtering. ***b***, Same format as in ***a*** but using resting-state MEG data from 15 participants. The circular histograms were collapsed across all phase conditions and participants. Individual participants’ accuracies and precisions in absolute phase distance are displayed for each phase condition, in which horizontal lines indicate mean values and error bars represent ± within-subject SEM.

Overall, the AKF algorithm outperformed the other two algorithms. For the synthetic data, the polar plots (after collapsing across the desired phases; the same pattern of results was found for individual phases; Fig. 3*a*, right panels) showed that the majority of the desired phases estimated by the AKF were close to the predetermined desired phases, as the phase distances between the two were densely clustered around zero. In contrast, the phases estimated by the other two algorithms were relatively scattered, as indicated by a higher number (i.e., the distance from the origin) of the estimated phases located away from zero. In support of this observation, both accuracy and precision indices (Fig. 3*a*, left panels) were improved when the AKF algorithm was used, and a shorter phase delay further enhanced the outcome.

For the resting-state MEG data from 15 participants, a similar pattern of results was found (the same pattern of results was found for individual phases; Fig. 3*b*). Performance was enhanced for all algorithms with a shorter phase delay (Harrison–Kanji test, main effect of phase delay, accuracy: *F*_(1,84)_ = 25.81, *p* = 0.0001, η_p_^2^ = 0.23; precision: *F*_(1,84)_ = 27.03, *p* = 0.0001, η_p_^2^ = 0.23). Performance among the algorithms was also significantly different in accuracy (main effect of algorithm, *F*_(2,84)_ = 64.00, *p* = 0.0001, η_p_^2^ = 0.60) and precision (*F*_(2,84)_ = 73.91, *p* = 0.0001, η_p_^2^ = 0.63). Further comparisons showed that for each phase delay, the AKF algorithm, relative to FFT, produced better phase-locked stimulations in terms of accuracy (Watson–Williams test, phase delay 45°: *F*_(1,28)_ = 8.94, *p* = 0.006, η_p_^2^ = 0.23; 180°: *F*_(1,28)_ = 7.00, *p* = 0.013, η_p_^2^ = 0.20) and precision (45°: *F*_(1,28)_ = 22.37, *p* = 0.0001, η_p_^2^ = 0.45; 180°: *F*_(1,28)_ = 19.56, *p* = 0.0001, η_p_^2^ = 0.40). Relative to AR, the AKF algorithm similarly showed better performance in terms of accuracy (45°: *F*_(1,28)_ = 80.94, *p* = 0.0001, η_p_^2^ = 0.73; 180°: *F*_(1,28)_ = 73.52, *p* = 0.0001, η_p_^2^ = 0.72) and precision (45°: *F*_(1,28)_ = 112.36, *p* = 0.0001, η_p_^2^ = 0.80; 180°: *F*_(1,28)_ = 88.56, *p* = 0.0001, η_p_^2^ = 0.75).

### The validity of the AKF algorithm during the real-time visual detection experiment

**Figure 4.**
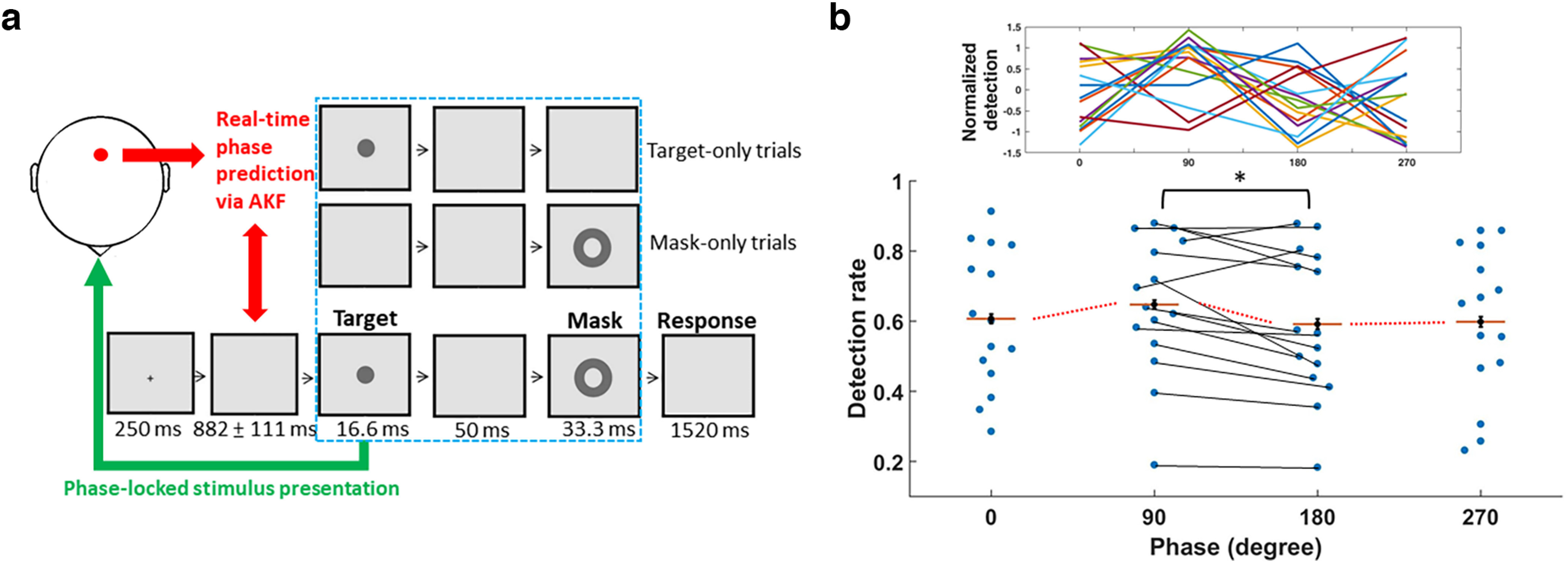
The design and behavioral results of real-time visual detection experiment. ***a***, Task design. Between the fixation and the target, a jittered blank screen (mean ± SD across participants) was naturally introduced by the analysis pipeline, reflecting its decision time for forward phase estimation. When the instantaneous phase derived from the predetermined posterior parietal MEG signal (red dot) approached the proximity of the desired phase (i.e., 180° of the phase delay from the future desired phase), the phase-locked target was presented at a given desired phase after accounting for the delay. Half of the trials contained both the target and mask (masked-target), a quarter of the masked-only trials had a blank screen in place of the target, and the remaining quarter of the target-only trials were without the mask. The stimuli were adjusted at the individual luminance threshold and thereby physically identical across trials. ***b***, Detection rates of the phase-locked masked targets as a function of the desired α phases. The red horizontal lines indicate mean values and are connected by the dotted lines. The profiles of the individual participants are depicted on the top, where the detection rates are *z* score normalized for visual comparison; *p < 0.05.

To investigate the direct role of the peristimulus α phase on visual detection, 15 new participants, as determined by an a priori power analysis, were instructed to detect the presence of a masked target that was implicitly presented at one of the desired phases in real time (Fig. 4*a*). Before the experiment, the luminance threshold of the background was established for each participant using a staircase procedure to converge a detection rate of ∼50% after five reversals. This phase-locked presentation was implemented by the AKF algorithm using MEG data from the same predetermined sensor (Fig. 4*a*, red dot), and the same pipeline was used (Fig. 2). Between the fixation and the target, a jittered blank screen was naturally introduced by the pipeline. The jitter mainly reflected that data were updated in chunks of 30 samples (30 ms or 1/3–1/4 α cycle on average; Fig. 2), and therefore, the pipeline was reinitiated several times because the phase delay between the instantaneous current and desired phases was frequently too short (<180°) to properly correct for the system-specific time lag. In this sense, this jitter simply indicated the “decision” time of the analysis pipeline and did not interfere with phase estimation per se.

Although our previous simulation results validated the PLSP approach with the AKF algorithm, we evaluated offline the extent to which the masked targets were successfully delivered at the desired phases to carefully confirm the performance of this approach in the real-time detection experiment. For this assessment, two analyses were adopted. First, we approximately reconstructed the phase at target onset using retraction-prediction analysis (see below). Second, we analyzed the mask-only trials offline to complement the first analysis. In the second assessment, because phase-locked triggers instead of targets were delivered in the mask-only trials, phases at trigger onset were extracted for analysis.

**Figure 5.**
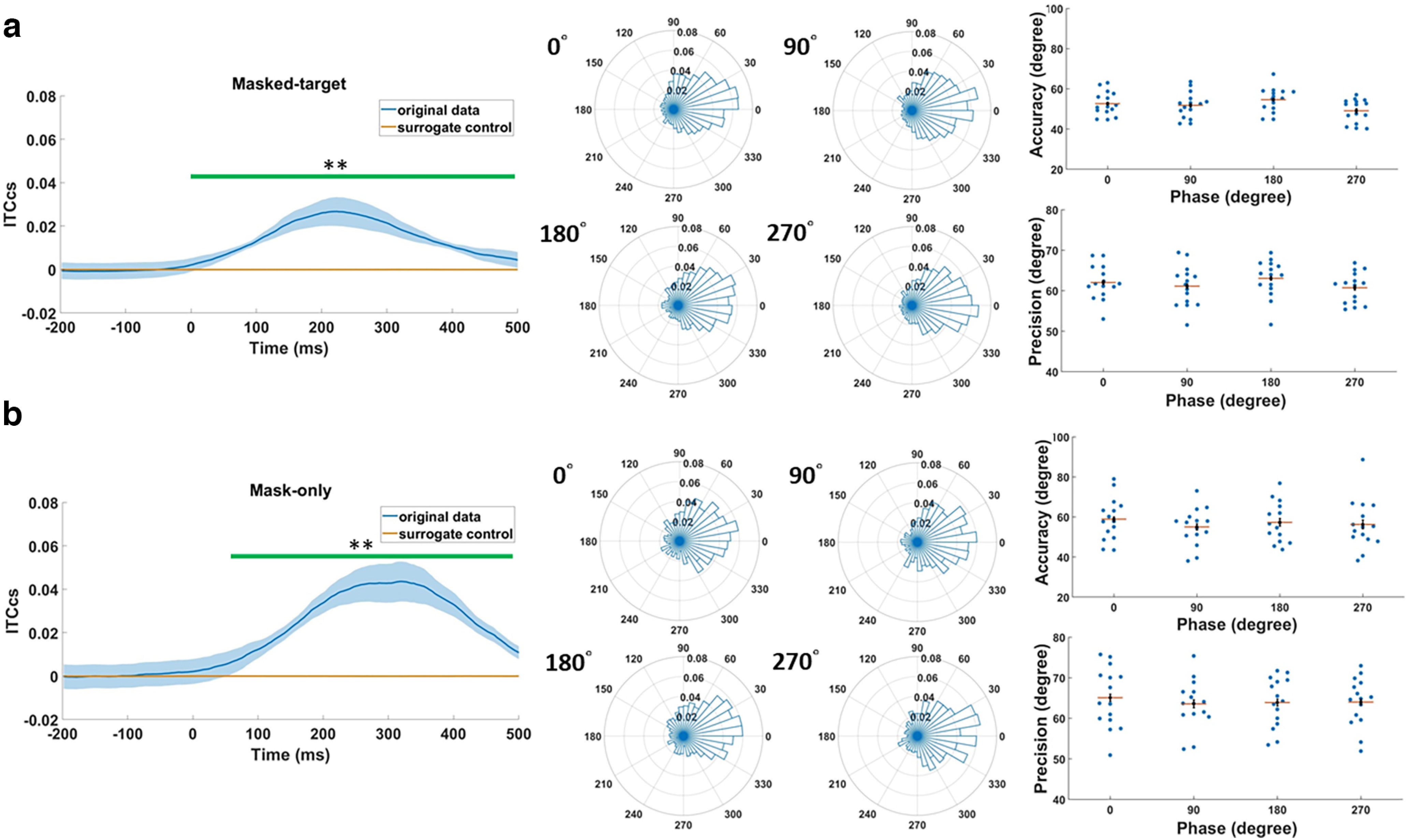
Assessment of the real-time adaptive Kalman filter (AKF) performance of phase-locked stimulus presentation (PLSP). ***a***, Real-time AKF performance of PLSP during the masked-target trials. The left panel shows the time courses of the cosine-similarity version of the intertrial phase coherence (ITC_CS_) during the masked-target trials from the predetermined sensor. The ITC_CS_ courses are plotted according to the original results and the surrogate control, which were created by computing the mean of surrogate ITC_CS_ values derived from the surrogate time series of the original data for 1000 repetitions. The green horizontal bar highlights the significant period. Shaded regions indicate ± within-subject SEM; **p < 0.01. The middle panels show the circular histograms of the absolute phase distances between the estimated and predetermined desired α phases collapsed across participants for each desired phase. The distance from the origin indicates the number of presentations falling within a bin. The right panels display individual participants’ accuracies and precisions in absolute phase distance as a function of the four phases. Red horizontal lines indicate mean values, and error bars represent ± within-subject SEM. ***b***, Real-time AKF performance of PLSP during the mask-only trials. Same format as in ***a***.

For the first analysis, if target presentation resets the phase dynamics, offline phase estimation at the target onset may have been rendered inaccurate because it could have been contaminated by the “smear” of the poststimulus phase effects elicited by the target because of the window-based/acausal signal analysis. Therefore, we first examined the presence of phase adjustment using the cosine-similarity version of the intertrial phase coherence (ITC_CS_) to mitigate the sample size bias (Chou and Hsu, 2018). After collapsing across phase conditions, increased ITC_CS_ was indeed observed relative to chance-level values (for details, see Materials and Methods, Surrogate data), which were created by computing the mean of surrogate ITC_CS_ values derived from the surrogate time series of the original data (cluster-based permutation test on −200–500 ms, *p* = 0.002 at approximately −8–500 ms, Cohen’s *d* for the average of the cluster = 0.79; Fig. 5*a*, left panel). To mitigate biased phase estimation at target onset because of the “smearing” effect caused by phase adjustment, we used past, uncontaminated instantaneous phase and frequency data for estimation. To determine which past data point could be safely used, we calculated the jackknife estimate of the onset latency of the phase reset based on the waveform between −50 and 500 ms, given that the cluster-based permutation tests did not indicate the true onset (Sassenhagen and Draschkow, 2019). The calculation gave rise to an estimated onset latency of 58 ms after target onset. Next, this estimated time point was traced 125 ms back (i.e., a cycle of 8 Hz from the onset latency of phase reset). The instantaneous α phase and frequency at −67 ms were derived by the bandpass-filter (8–12 Hz) Hilbert transform and were used to forecast the phase at target onset. Next, we formally applied the above retraction-prediction analysis to the masked-target trials to estimate the phases at target onset. Similar to our previous simulation results (Fig. 3*b*), real-time target presentation was largely concentrated around the desired phases (Fig. 5*a*, right panel), and algorithm performances did not significantly differ across the phases (Watson–Williams test, accuracy: *F*_(3,56)_ = 2.14, *p* = 0.11; precision: *F*_(3,56)_ = 0.83, *p* = 0.48).

For the second complementary analysis, we analyzed the mask-only trials offline. The “smearing” effect caused by the phase reset, in terms of the ITC_CS_, was less severe during the mask-only trials because there was no target presentation and the mask was presented 66.6 ms later (cluster-based permutation test, *p* = 0.002 ∼54–500 ms, Cohen’s *d* for the average of the cluster = 0.86; Fig. 5*b*, left panel). The jackknife estimate of the onset latency of this phase adjustment was 102 ms after the target onset based on the waveform between −50 and 500 ms. Given that this time point was approximately one α cycle away from the target onset, the peristimulus phase could be reasonably extracted in this trial condition. Our complementary evaluation confirmed that the triggers were mostly phase-locked to the desired phases (Fig. 5*b*, right panel). Moreover, as before, the performance was not significantly different across all phase conditions in terms of accuracy (*F*_(3,56)_ = 0.36, *p* = 0.78) and precision (*F*_(3,56)_ = 0.16, *p* = 0.92). Altogether, our converging results validate the AKF algorithm in the implementation of the phase-locked presentation during the real-time visual detection task, primarily based on the simulation results (Fig. 3) together with supporting offline analyses of the masked-target (Fig. 5*a*) and masked-only trials (Fig. 5*b*).

### The behavioral effect of the α phase on the visual detection of masked targets

For the real-time visual detection experiment, the average detection rate (the proportion of detected targets or hits) on the masked-target trials was 61% (SD = 19%, range = 31–88%). While detection on the target-only trials was 96% (SD = 2%, range = 85–99%), which reflected the actual detection of the target, the false alarm rate on the mask-only trials was low (mean: 16%, SD = 12, range = 1–43%). Signal detection analyses indicated an average criterion of 0.42 (SD = 0.52, range = −0.23–1.68) and an average d′ of 1.4 (SD = 0.66, range = 0.21–2.65). None of the participants were excluded according to the exclusion criterion (detection > 90% or < 10%, false alarm > 85%) used in a previous study on masked-target trials with a similar design (Mathewson et al., 2009).

Based on the validity of the phase-locked presentation, the following analysis path was adopted to examine the phase-dependent behavioral effect. In contrast to most of the previous studies based on the offline correlation approach, here the peristimulus phases were the independent instead of dependent variable and thereby were predetermined a priori. Because four levels of the phases were chosen because of the limitation of current real-time PLSP, we calculated the detection rate for each desired phase and then examined whether there was differential enhancement on detection. The result showed that the overall detection rates did not significantly vary (one-way repeated-measures ANOVA, *F*_(3,42)_ = 2.47, *p* = 0.075, η_p_^2^ = 0.15, achieved power = 0.96; Fig. 4*b*).

### Exploratory analysis of the α phase-dependent behavioral effect

Despite the null main effect, enhanced detection associated with the 90° desired phase seemed to be especially evident. The follow-up comparisons confirmed this exploration, as the 90° detection rate was significantly higher than the 180° detection rate (two-tailed Dunnett’s multicomparison test to check whether there was enhanced detection at 90° (Howell, 2010), *p* < 0.05, Cohen’s *d* = 0.69) but not the others (all *p*s > 0.05). With respect to the false alarm, criterion, or d′, no significant variations were observed according to the phase conditions (*F*s_(3,42)_ ≤ 1.864, *p*s ≥ 0.15) after *post hoc* comparisons between the 90° and 180° phases (all *p*s > 0.05).

Although the magnitude of the mean difference in detection (5.6%) between the 90° and 180° phases was comparable to the previous results (Mathewson et al., 2009), to further verify that the finding could be attributable to the current phase-locked performance, a permutation procedure was adopted. Specifically, we pooled the trials from both phase conditions for individual participants. The trials were randomly selected without replacement and reassigned to one of the four phase conditions. Next, we recomputed the detection rates and the corresponding two-tailed paired *t* tests. This procedure was repeated 1000 times to generate a resampling distribution of t statistics that was derived from different combinations of phase-locked presentations. The t statistic of the original data were larger than 99% of the samples of the distribution, confirming the significance of the finding.

### Exploratory analysis of the neural dynamics underlying α phase-dependent visual detection

**Figure 6.**
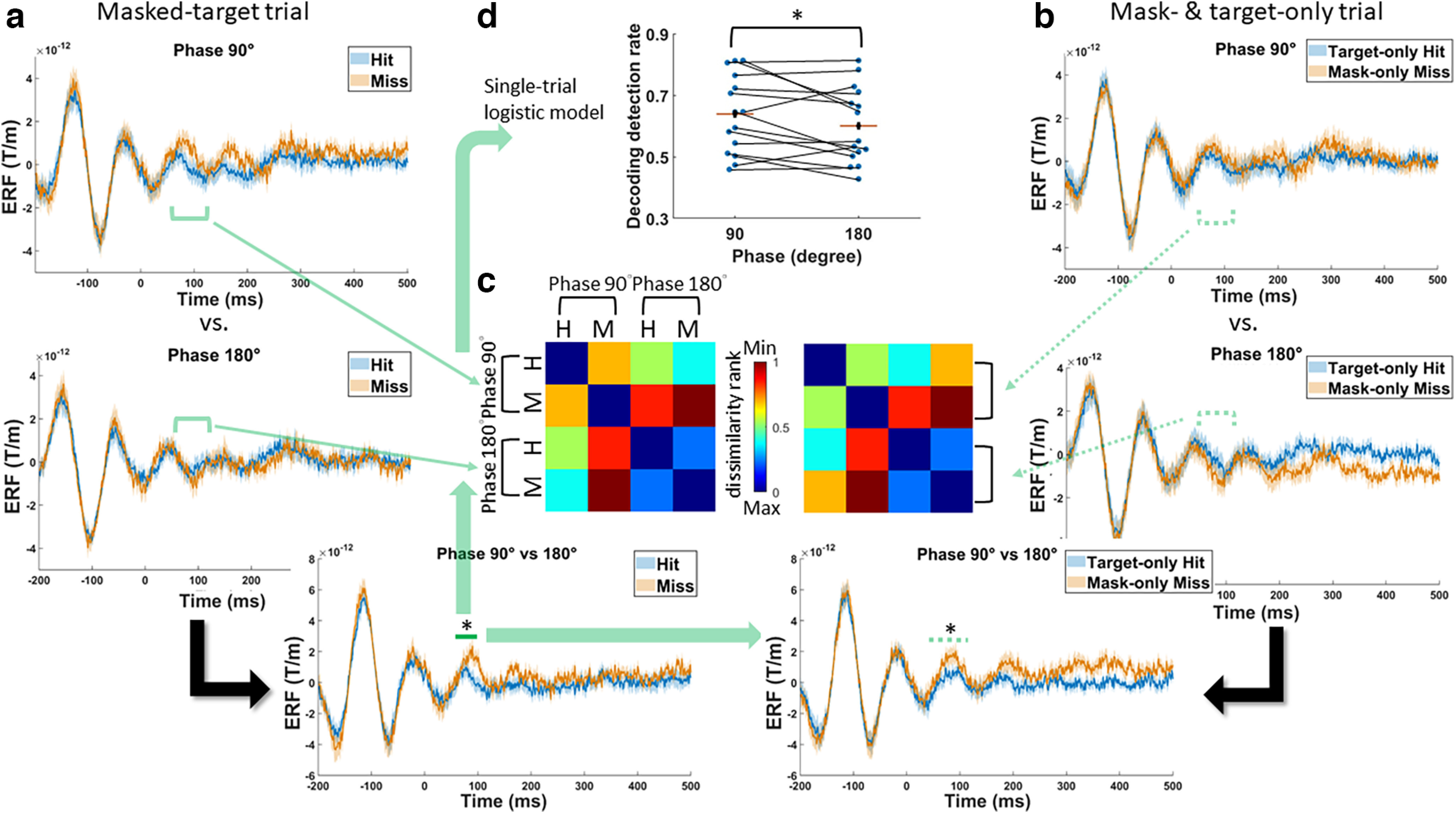
The neural dynamics underlying phase-dependent visual detection. ***a***, Time courses of the event-related fields (ERFs) during the masked-target trials from the predetermined parietal sensor. The ERFs are plotted according to the behavioral outcome (hits vs misses) and the desired phase (90° vs 180°). The green horizontal bar highlights the significant period for the interaction effect. Shaded regions indicate ± within-subject SEM; *p < 0.05. ***b***, Same format as in ***a*** but using the target-only miss trials and the masked-only hit trials. The green dotted horizontal bar highlights the significant interaction based on the period identified from the masked-target trials. ***c***, Subject-averaged representational dissimilarity matrices of the ERFs based on hits (H)/misses (M) and phases from the masked-target trials (left panel) or from the mask-only and target-only trials (right panel). The matrices were constructed by calculating cosine similarity between the ERFs during the significant interaction period (horizontal green solid or dotted line). Each matrix is separately rank-transformed and scaled into [0,1]. ***d***, Decoding the relevance of the data during the significant interaction period in shaping behavioral outcomes. A logistic regression classifier was separately trained to distinguish hit trials versus miss trials based on single-trial MEG activity recording from the 90° or 180° desired phase. Classifier output on test trials produced higher decoding detection during the 90° relative to 180° phases after repeated stratified cross-validation; *p < 0.05.

Our *post hoc* behavioral findings were accompanied by early changes in underlying brain activity. Guided by the offline behavioral findings (i.e., the difference in detection between the 90° and 180° desired phases), we calculated the event-related fields (ERFs) on the predetermined sensor according to the hit or miss trials for the 90° and 180° desired phases separately. In agreement with our experimental manipulations, before the target onset (Fig. 6*a*), the ERFs oscillated within the α range with a phase shift of 131.58 ± 36.00° between the 90° and 180° phase conditions (sinusoidal fitting on the baseline between −200 and 0 ms collapsed across participants and behavioral outcomes, 90°: 9.57 ± 1.31 Hz, *R*^2^ = 0.69 ± 0.15; 180°: 8.69 ± 0.59 Hz, *R*^2^ = 0.73 ± 0.12). Immediately after target onset, oscillatory patterns appeared to bifurcate between the two trial types during the 90° phase, as indicated by the elevated ERFs of the misses, but not during the 180° phase. A significant interaction effect was found in the early ERFs (cluster-based permutation test on 0–500 ms, *p* = 0.024 ∼83–102 ms, Cohen’s *d* for the average of the cluster = 0.93; Fig. 6*a*). Next, jackknife estimates were calculated to rigorously establish effect latency based on the waveform between 50 and 150 ms. This calculation gave rise to a grand-averaged estimate of 70 ms for onset latency and 121 ms for offset latency. Within this identified latency, additional analysis confirmed that the obtained effect reflected the influence of the α phase per se rather than the additional interference from mask processing. When subtracting the ERFs of the mask-only trials from those of the corresponding masked-target trials, the residual ERFs still showed a significant interaction effect (two-way repeated-measures ANOVA on phase × behavioral response, *F*_(1,14)_ = 7.94, *p* = 0.014, η_p_^2^ = 0.36). In addition to the interaction, we examined whether there was a main effect of phase or behavioral response on ERFs after collapsing data across the irrelevant dimension. After subtracting the ERFs of the mask-only trials to account for phase shifts between the phase conditions, no significant main effect was found (phase: *p* = 0.062; behavioral response: *p* = 0.24). In summary, the observed interaction effect indicates that the information processing of the same target differed depending on the peristimulus phases, which yielded different temporal dynamics for hits and misses.

We further checked to what extent the interaction effect was related to the processing of incoming targets and to resulting behavioral outcomes. With respect to the first issue, we investigated how the previously observed interaction would behave when the stimulus condition differed, such as during the mask-/target-only trials. If a similar phenomenon is observed, this would indicate that the interaction is likely to reflect a general representation of information processing across stimulus dimensions. First, a high similarity was found in the interaction effect between the early ERFs of hits and misses from the masked-target trials and those from the mask-/target-only trials; the former dataset served as a reference for comparison. Specifically, when the ERFs of two phase conditions were compared, a significant interaction during the effect latency was similarly observed between the target-only “hit” trials and the mask-only “miss” trials (*F*_(1,14)_ = 6.65, *p* = 0.023, η_p_^2^ = 0.32; Fig. 6*b*). To provide a global pattern of such relatedness, for each participant, we calculated the cosine similarity between every pair of early ERFs (70–121 ms) to construct two 4 × 4 (2 phase × 2 behavioral outcome) RDMs for the masked-target and mask-/target-only trials (Fig. 6*c*). These two RDMs were then compared (Kendall’s rank correlation; τ_a_ = 0.27), and significant relatedness was found (one-tailed signed-rank test, *p* = 0.016). Given that the phase-modulation patterns were similar regardless of the stimulus conditions, these findings not only confirmed that the different patterns of the response-related ERFs depended on the phases (i.e., similar phase-dependent modulation using different sets of trials) but also revealed that the response-related ERFs represented the fate of phase-dependent perceptual rather than stimulus processing (i.e., similar phase-dependent modulation when the stimulus condition differed). This led us to test whether for the masked targets, information content at the effect latency was differentially predictive of ultimate behavioral outcomes according to the α phase. The single-trial data from 70 to 121 ms were fitted to a logistic regression separately for each phase condition to distinguish hits from misses. Through 10 repeated stratified fivefold cross-validation, the obtained decoding detection was enhanced during the 90° phase relative to that in the 180° phase, thus reproducing the previous behavioral pattern (one-tailed *t*_(14)_ = 2.02, *p* = 0.032, Cohen’s *d* = 0.52; Fig. 6*d*). All these results collectively revealed that α phases differentially interacted with the incoming masked targets, resulting in early phase-modulated neural representations pertaining to the perceptual fate of those stimuli, which was, in turn, relevant to biasing eventual detection rates.

### The control analysis of the power and instantaneous frequency effect

Given that α power may affect corresponding phase estimation and that prestimulus power is also thought to be involved in visual detection (Benwell et al., 2017; Zazio et al., 2022), we examined whether our phase-dependent behavioral and neural results could be explained by concurrent power changes. First, at the behavioral level, the detection difference was likely because of the phase effect, as there was no significant power difference between the 90° and 180° phases around or before target onset (cluster-based permutation test on −50–50 or −500–0 ms, *p* = 0.15 or 0.18). Moreover, the observed ERF effect was not accompanied by corresponding power changes, as no significant interaction (phase × behavioral outcome) in power was observed after target onset (*p* = 1). We also divided the trials into high-power and low-power bins using a median split for each phase condition. For both high-power and low-power trials, no significant prestimulus or peristimulus power difference was observed between the phases (all *p*s > 0.15). We also examined whether our results could be explained by the changes in the prestimulus instantaneous frequency (Samaha and Postle, 2015). Similarly, no significant difference in the instantaneous frequency was found between the 90° and 180° phases around or before target onset (*p* = 0.44 or 0.13).

### The generalization of phase-dependent neural modulation

**Figure 7.**
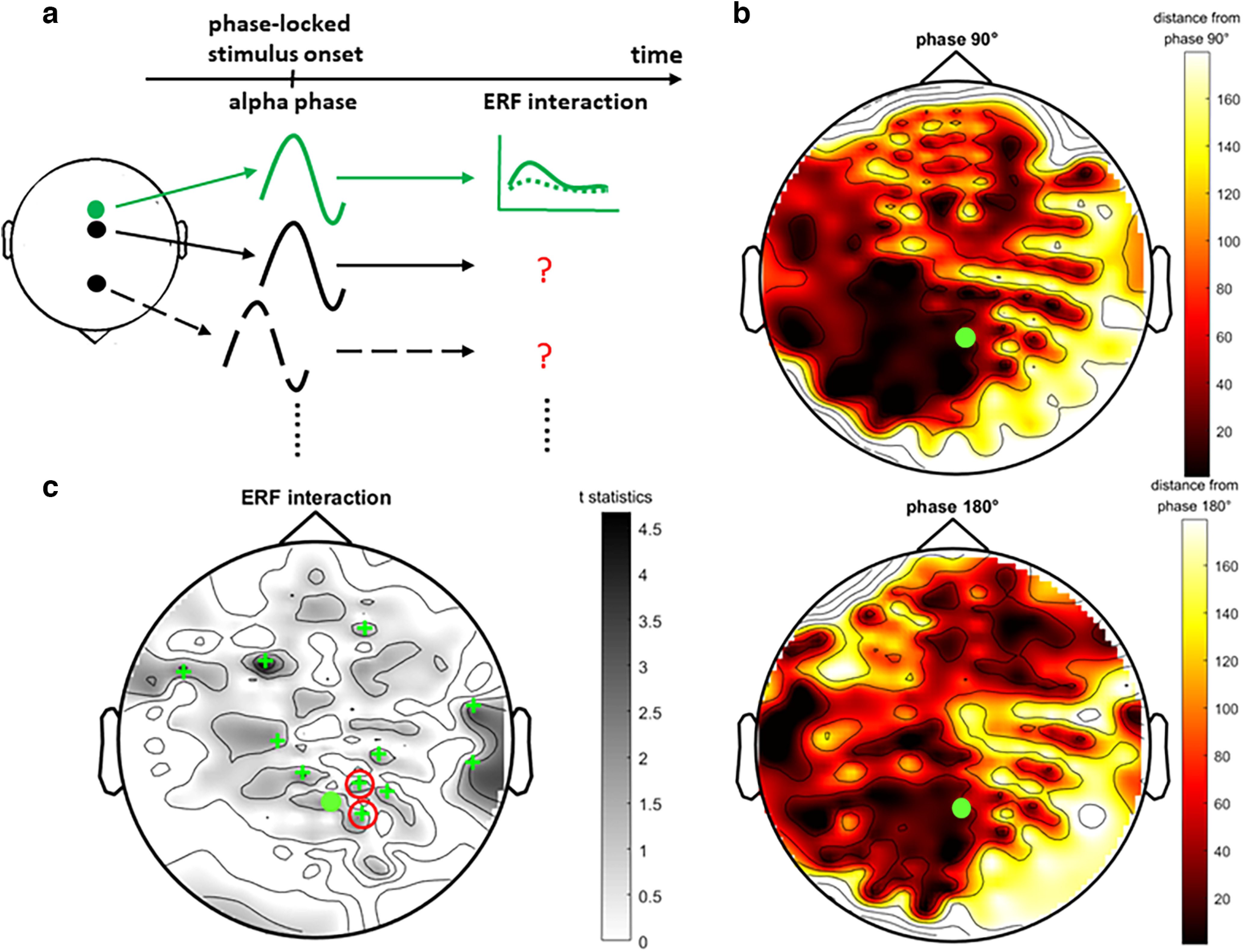
The generalization of phase-dependent neural modulation. ***a***, Schematic of the analysis flow. The green diagram represents the analyses that were conducted on the predetermined sensor, whereas the black diagrams represent the remaining sensors with similar or dissimilar phase-locked behavior to be examined. ***b***, Scalp topographies of the circular means of the absolute phase distances from the 90° (top panel) and 180° (bottom panel) desired phases at target onset after collapsing across participants. The green solid circles indicate the predetermined posterior parietal sensor (*p* < 0.05). ***c***, Scalp topographies of the event-related field (ERF) interaction on the hit-miss differences between the 90° and 180° desired phases during the effect period (70–121 ms). The green crosses highlight the sensors exhibiting significant interactions. Among those sensors, the red circles highlight the ones whose single-trial peristimulus phase distributions closely matched that of the predetermined sensor (*p* < 0.05).

We proceeded to explore whether the aforementioned phase-dependent ERF interaction effect was similarly observed at the other sensors during the masked-target trials where stimulus presentation was locked to the same or different phase from that of the predetermined sensor (Fig. 7*a*). For this analysis, we first estimated how the peristimulus α phases at these sensors deviated from that of the predetermined sensor. We calculated the circular mean for the peristimulus phases across trials at every sensor based on the retraction-prediction analysis outlined above. As depicted in Figure 7*b*, the results are reported in terms of the absolute phase differences from the corresponding desired phases (90° or 180°) after collapsing across participants. A spot with a darker color (i.e., the hotspot of the phase topography) indicates that the mean peristimulus phase is more similar to that of the predetermined sensor. In general, for both conditions, α oscillations, predominantly at the left occipito-temporal-parietal sensors together with the predetermined sensor (Fig. 7*b*, green solid circle), traveled approximately in synchrony and simultaneously neared the desired phase angles at target onset. Next, some sensors, mainly in the posterior parietal region, exhibited a similar pattern of previously observed ERF interaction during the effect period (70–121 ms; one-tailed paired *t* test on hit-miss differences between phases given the directionality of interaction, *t*s_(14)_ mean ± SD = 2.41 ± 0.76, *p*s mean ± SD = 0.024 ± 0.015, Cohen’s *d* mean ± SD = 0.62 ± 0.21; Fig. 7*c*, green cross). Among the sensors exhibiting a significant interaction, most seemed to fall within the hotspots of the phase topographies when comparing Figure 7*b*,*c*. A detailed examination designed to provide additional support showed that the single-trial peristimulus-phase distribution of two sensors (Fig. 7*c*, red circle), near the predetermined sensor, significantly resembled the distribution of the predetermined sensor at both 90° (circular correlation on individual subjects, ρs mean ± SD = 0.39 ± 0.10, *p*s mean ± SD = 0.002 ± 0.009 for 14/15 subjects) and 180° desired phases (ρs mean ± SD = 0.36 ± 0.10, *p*s mean ± SD = 0.003 ± 0.006 for 13/15 subjects). Taken together, our previous neural findings from the predetermined sensor were not a random effect but were attuned to our experimental manipulation and could be generalized to other posterior parietal sensors for which the stimulus presentation was locked to similar desired phases.

## Discussion

**Figure 8.**
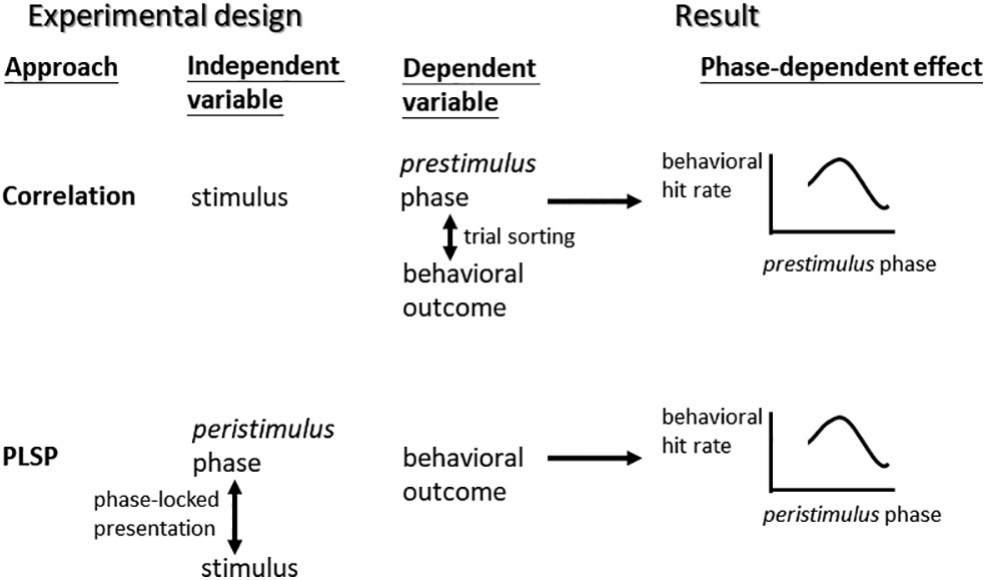
A schematic comparison between the previous correlation and current phase-locked stimulus presentation (PLSP) approaches. Notably, although both the PLSP and correlation approaches produce the relationship between phases and behavioral outcomes, the generative processes underlying this relationship are different.

Beyond previous research, this study capitalized on the real-time PLSP approach that was implemented by our newly developed phase-estimation algorithm. By evaluating the phase as an independent variable (Fig. 8), this new approach allowed us to investigate whether and how the endogenous critical α phases, during which masked targets were simultaneously presented, directly led to behavioral and neural changes in visual detection. We first confirmed that our algorithm could deliver the target stimuli around the desired phases and perform better than the existing algorithms. The overall change in detection according to the peristimulus α phase was insignificant. Nevertheless, *post hoc* analyses seemed to reveal the presence of phase-dependent visual detection to some extent. Compared with previous positive evidence, these discrepancies might reflect several differences in methodology and analysis, as discussed below.

### The performance of the AKF algorithm

One objective of this study is to develop a robust phase-estimation algorithm to implement real-time PLSP. From the perspective of the phase estimation algorithm, accurate estimation of the real-time phase is hindered by several factors: phase singularity because of the presence of noise or low spectral power in the raw data, the edge effect (i.e., distortion at the ends of the processed data segment) and, relatedly, the issue of causality (i.e., data required before and after the time point of interest) that are relevant to filtering and spectral analyses (Hurtado et al., 2004; Shirinpour et al., 2020). The latter especially poses a serious issue, as during real-time implementation, data are always recorded until the current time point, and future data are not available. Furthermore, the obtained instantaneous phase at the current moment actually indicates the near past phase because of a phase/time delay that results from a system-specific time lag caused by data, hardware (including stimulation), and real-time sampling processing. A robust algorithm is therefore required to forward predict the future desired phase based on the precise estimation of the instantaneous phase and frequency (phase rate) at the current time (Fig. 1*b*). In turn, PLSP can be implemented with acceptable accuracy under real-time constraints. In the present experimental setting, the newly introduced AKF algorithm performed more robustly than the common AR and FFT algorithms. The core of all three algorithms serves the same purpose, i.e., ensuring that the instantaneous phase and frequency at the current time point can be accurately estimated to forecast the future desired phase.

However, for the AR algorithm, signal noise, which is often high in electrophysiological recordings, is included for computation. This result might explain its inferior performance (Mansouri et al., 2017). For AR to perform at its best, a somewhat exhaustive empirical search is usually required to obtain the best combination of hyperparameters beforehand for individual datasets and participants. Nevertheless, this time-consuming process was not conducted in this study because of the time constraint and the need for a consistent and practical analysis pipeline for algorithm comparison. Regarding the FFT algorithm, because the whole signal is reduced to a simple sine function, this simplification may have impaired performance (Shirinpour et al., 2020), especially given that the MEG signal of interest here was relatively broadband (8–12 Hz for α) in the sense that the signal was nonstationary and composed of several frequency components.

In contrast to the above two algorithms, the AKF algorithm performs recursive and adaptive Bayesian-like computation by estimating the current state by scaling the information from both the predicted states and noisy measurements. Through this process, the edge effect and phase singularity could be mitigated, resulting in better estimations than those based on measurements alone. However, our algorithm has room for further improvement. For example, the phase-locked stimulus presentations between the desired phases somewhat overlapped, which might bias the current results. A more intricate transition function or a hardware upgrade (e.g., a higher refresh rate of the projector) in the future could help to better capture the phase dynamics or reduce the phase delay (Fig. 3), which may enhance the performance of phase-locked presentation.

### The effect of the peristimulus phase on visual detection as revealed by real-time PLSP

Regarding the second objective of this study, the peristimulus phase does not seem to robustly enhance visual detection. From a methodological perspective, in the majority of previous research, the widely adopted correlation approach is informative but constrained (Fig. 8). In addition to failing to make directional inferences about the role of the α phase, evidence is primarily built on the relationship between behavioral performance and the prestimulus α phase. However, fluctuations in the ongoing α phase have been linked to the effect of breathing (Hsu et al., 2020) or expectation (Samaha et al., 2015). These confounding variables not only complicate the interpretation of the results but may also contaminate the data and thereby yield mixed evidence. Most importantly, the poststimulus phase activity elicited by the stimulus may crossover into the peristimulus window, thus obscuring offline examination of the effect of the α phase around stimulus onset (Brüers and VanRullen, 2017).

To address the above issues, the current study employed the PLSP approach to investigate how α peristimulus instead of prestimulus phases directly affected the perception of simultaneously presented stimuli. A few previous studies have already employed the PLSP approach to investigate the phase effect on the reaction time. In a recent study (Vigué-Guix et al., 2022), stimuli [light-emitting diode (LED) flashes] were presented online at specific ongoing α phases using the FFT algorithm, but in contrast to earlier work (Callaway and Yeager, 1960; Dustman and Beck, 1965), negative evidence was reported. In line with this recent study, current evidence also fails to fully support the presence of a phase-dependent behavioral effect, as indicated by the null main effect despite the borderline significance (*p* = 0.075) and a large effect size (η_p_^2^ = 0.15). One possible reason is that our sample size was derived from a prior study using a similar design (Mathewson et al., 2009); however, distinct from this key study, the current study jittered the timing of target presentations to preclude temporal expectation. This change in the design together with a different analysis path being followed, as discussed below, might bias the derived effect size, and therefore, a larger sample size might be required to reach a robust effect. Overall, although the PLSP approach provides a different perspective on the debate regarding the phase effect, a full understanding of this issue may still require the future development of multifaceted techniques and analyses.

From an analytical perspective, in regard to previous positive evidence, trial sorting is commonly conducted to derive the prestimulus phase on a trial-by trial basis to establish its relationship with the associated behavioral responses (Fig. 8). Is it possible to derive the peristimulus phase based on the prestimulus phase using our retraction-prediction analysis, which was performed to estimate the peristimulus phases of the masked targets offline on the predetermined sensor to validate real-time phase-locked implementation (Fig. 5*a*) and on the remaining sensors to examine the generalization of phase-dependent neural dynamics (Fig. 7)? However, the utility of the retraction-prediction analysis only provides an indirect approximation regarding whether the overall peristimulus phases cluster around a given desired phase, i.e., the focus of our analyses. If the offline peristimulus phases are estimated in this manner, their validity on a trial-by-trial basis still cannot be verified because the original peristimulus phases are unlikely to be backwardly recovered for the reason outlined above. Accordingly, the current study examined the phase-dependent behavioral effect based on four predetermined desired phases, and no trial sorting was performed. This analysis strategy nevertheless leads to coarse sampling of the full α cycle, and in turn, a more detailed pattern of the phase effect cannot be revealed. Such a limitation, together with the possibility that the “preferred” phase (i.e., the phase associated with enhanced detection) differs across participants, might offer a possible explanation regarding the null main effect of the peristimulus phase on detection.

### The *post hoc* behavioral and neural results

Our follow-up and less conservative behavioral analyses (i.e., limiting our questions to a relatively few comparisons) nevertheless revealed the potential modulatory role of the peristimulus α phase in visual detection. These results showed that the phase, especially at 90° relative to 180°, likely directly enhanced the detection of the masked targets. Such behavioral evidence was corroborated by the corresponding neural findings which provided a new perspective on the underlying mechanism. Between those two angles, the ERFs associated with hits and misses exhibited different patterns at an early stage. This observation echoes prior literature indicating that the α phase affects early event-related components induced by stimuli (Makeig et al., 2002; Mazaheri and Jensen, 2010; Fellinger et al., 2011). As the targets were physically identical across trials and the ERF effects occurred regardless of the stimulus conditions, the above early phase-dependent neural signature likely manifested the perceptual fate of the incoming masked targets that resulted from the interaction between the α phases and the targets, which in turn potentially played a role in shaping the eventual detection outcomes. All these findings cannot be simply explained by concurrent changes in α power or confounding factors in phase-locked stimulation performances between the phase conditions. Notably, the neural signature was specifically observed in the posterior parietal region (Fig. 7). In line with our findings, the parietal region is commonly activated in visual masking protocols (Dehaene and Changeux, 2011) and contains early information needed for distinguishing different percepts (Salti et al., 2015). Notably, the detection changes did not exhibit an ideal oscillatory pattern such that detection differs between the adjacent angles but not at the opposite angles (however, see (Menétrey et al., 2022) and (White, 2018) for a discussion on whether the phase-dependent behavioral changes are guaranteed to be cyclic). This finding might reflect coarse sampling of the full α cycle, and in turn, a more detailed pattern of the phase effect cannot be revealed, as discussed above.

From a conceptual perspective, the current neural evidence offers new insights into the underlying mechanism. According to a previous notion, the α phase represents ongoing fluctuations in cortical excitability (Lindsley, 1952; Fries, 2005; Klimesch et al., 2007). The perceptual fate of a visual stimulus thus depends on the instant that it coincides within the excitability of the ongoing α cycle, which produces alternations of hits and misses between more favorable and less favorable phases (Varela et al., 1981; VanRullen, 2016). Accordingly, most previous research has directly explained the phase-dependent behavioral pattern as reflecting alternations of the perceptual states according to the phase. At the neural level, this notion would expect an overall difference (i.e., regardless of behavioral outcomes) in early activity between the phases (Mathewson et al., 2009). However, during the excitable or less excitable phase, the stimuli are not always detected or undetected. What is the neural fate of these stimuli? Given that few studies have investigated the resulting neural changes, here, we delved deeper into this issue and found that the early ERFs varied according to both the phases and the behavioral outcomes (Fig. 6*a*). This interaction pattern is consistent with the patterns observed in the power-dependent neural effect (Zazio et al., 2022), for which the differences between the “hit” and “miss” associated event-related activities vary according to the high and low prestimulus power conditions.

Given that the idea of alternations of the perceptual states cannot fully address the complex interaction effect observed here and the phase effect is not as robust as previously presumed given that negative evidence (including the current null main effect) has accumulated, we propose a revision of this notion. Our proposition suggests that the peristimulus α phase interacts with incoming stimuli, and then this interaction orchestrates the neural representation of the perceptual fates of the stimuli at an early stage. This neural representation varies according to the phases and possibly constitutes a factor that biases the later decision-related processes and eventually the behavioral outcomes. In other words, at a given peristimulus phase, the same target is processed in such a manner (increasing neural fluctuations for instance) that its information content could facilitate detection. Accordingly, in addition to the peristimulus phase per se that could alternate incoming perceptual events by providing different temporal frames (Varela et al., 1981; VanRullen, 2016), the phase might further interact with stimuli by differentially biasing their information processing. In this view, the stimulus properties, perhaps including the task requirements that are relevant to early information processing, may have equally contributing roles to determine the magnitude of phase-dependent perception.

To conclude, using real-time PLSP, stimuli can be presented as a function of the simultaneous α phase, which enables more direct experimental control for allowing directional inference about the extent to which rhythmic modulation in perception is a direct result of the underlying brain oscillations. Together with the phase-estimation algorithm shown here, the current approach may be used as a practical tool for studying the functional role of ongoing phase activity to better understand how the brain state impacts cognitive processing.

## Synthesis

Reviewing Editor: Christoph Michel, Universite de Geneve Decisions are customarily a result of the Reviewing Editor and the peer reviewers coming together and discussing their recommendations until a consensus is reached. When revisions are invited, a fact-based synthesis statement explaining their decision and outlining what is needed to prepare a revision will be listed below. The following reviewer(s) agreed to reveal their identity: Jelena Trajkovic.

Unfortunately the second reviewer that was secured for this manuscript had to withdraw his agreement due to personal problems. In order to avoid further delay and given the fact that the one reviewer gave very detailed and competent comments I decided to base the decision on this one reviewer.

I attach the comments of the reviewer here and ask to respond to each of the point raised and indicate the changes made in the manuscript in response to the questions.

Reviewer’s comments:

The article entitled “The effect of the peristimulus alpha phase on visual perception through real-time phase-locked stimulus presentation” tackles a much-investigated (but still inconclusive) role of the phase of alpha activity in determining perceptual outcomes. To this aim, the authors approach the problem from a new perspective: instead of determining the alpha phase of the hits vs misses post hoc, they develop a novel algorithm able to predict the alpha phase, and thus present the visual stimuli on specific alpha phases (here 0, 90, 180, 270 degrees), and subsequently look at the behavioural differences between them. First, they convincingly demonstrate that the algorithm is successful in correctly predicting the phase and presenting the stimulus at that exact phase. Second, they demonstrate that, although the overall differences across phases were not significant, looking at the opposite phases (90 vs 180 degrees) yielded significant differences in hit rates. Third, they investigate the interaction between the alpha-phase and ERF brought by the stimulus, which suggested an interaction between the phase and the information brought by the stimulus itself, which seems to explain the visual accuracy. Based on these results, the authors conclude that the phase itself cannot entirely explain the perceptual outcome that rather depends on the interaction between the phase and the stimulus.

The article is well written, and I appreciate the advanced methodology used and the extensive results exploration. Likewise, the algorithm that the authors developed and its validation could be useful to a broad research community aiming at sensory or neural stimulation at the exact, online-estimated oscillatory phase. Finally, the results are intriguing and go well in hand with previous findings on the topic. That being said, I also have several concerns, questions and suggestions that would further aid the quality of the article.

MAJOR POINTS:

1. I am wondering if the results could be influenced by the within- and between participants differences in the value of the pre-stimulus instantaneous alpha-frequency. Specifically, my point relates to the fact that the length of an alpha cycle differs within the alpha range (i.e., for 8 Hz the cycle lasts for 125ms and for 12 Hz 83ms), and if we are aiming for the quarter of a cycle, which is the phase resolution chosen here, we are talking about 31 vs 20 ms for 8 and 12 Hz, respectively. Given that the refresh rate of the monitor used for the stimuli presentation is 60 Hz, leaves us with a potential marginal error of 16 ms. Now, the problem that I perceive is that this marginal error would be different for low vs high alpha, resulting in more precise phase estimation, thus even phase targeting, for lower vs higher alpha frequencies. Could the authors control if this is the case, or comment why this shouldn’t matter?

2. Related to the first point, recent findings have also demonstrated a direct role of alpha-frequency in perceptual sampling and accuracy. I would suggest the authors to also test if this could be the case here (possibly explaining the marginally significant effect of the phase).

3. The authors have conducted some control analysis regarding pre-stimulus alpha power: specifically, they looked at power differences between the opposite phases (90 vs 180 degrees). However, one of the prominent theories in the literature (pulsed-inhibition theory) is that there is a pre-stimulus alpha power-phase trade-off, where different levels of prestimulus alpha amplitude will dictate the effects of the phase-differences. Therefore, I would suggest the authors to look at the differences between the four phases separately for high vs. low alpha-amplitude trials.

4. A marginally significant effects of the main ANOVA analysis, together with the description of how number of participants was determined, makes me wonder if the effect hasn’t reached significance due to underestimation of the sample size. If I understood correctly, the expected effect size was estimated from a t-test conducted in a previous study? However, the main analysis here was 4 factor ANOVA (which was marginally significant) and not the comparison between the two conditions (which, in the end, was actually significant). Can you please comment on this possibility?

5. I feel that the conclusions in the discussion section about the phase effects vs. phase-stimulus interaction effects are too far-fetched and more speculative, rather than definite, based on the obtained results. First, this analysis was exploratory, as claimed by the authors. Second, the phase results are already marginally significant, as well as the main effect of phase for the ERFs (0.062), that, in my opinion, cannot convincingly exclude the relevant role of the phase itself. Third, a number of factors could influence the ERFs themselves, such as pre-stimulus power (as previously demonstrated: Zazio et al., 2021). Therefore, the phase-ERF interaction may not be as straightforward as presented here. All these reasons make me wonder if the authors should be more cautious when proposing this phase-stimulus interaction as a possible main predictor of perceptual accuracy.

MINORS

1. Line 8: based on adaptive Kalman filtering. This information guided online presentations of masked stimuli

2. Line 10: Detection rate instead of detection hit

3. Line 33: which is involved in instead of involves in

4. Line 479: resets instead of reset

5. Line 537-538: I would be careful when saying that: Given that post hoc tests are valid even if the overall ANOVA does not find a significant difference among means,... as I don’t think this is an overall shared opinion in the statistical community. However, as the main ANOVA effect is marginal, and you already describe this analysis as exploratory, I don’t have anything against it, just don’t think that you necessarily need to justify it in this way.
